# The effectiveness of low-level diode laser therapy on orthodontic pain management: a systematic review and meta-analysis

**DOI:** 10.1007/s10103-015-1743-4

**Published:** 2015-03-24

**Authors:** Chong Ren, Colman McGrath, Yanqi Yang

**Affiliations:** Faculty of Dentistry, The University of Hong Kong, 34 Hospital Road, Sai Ying Pun, Hong Kong SAR, China

**Keywords:** Low-level laser therapy, Diode laser, Orthodontic pain, Systematic review

## Abstract

To assess the effectiveness of diode low-level laser therapy (LLLT) for orthodontic pain control, a systematic and extensive electronic search for randomised controlled trials (RCTs) investigating the effects of diode LLLT on orthodontic pain prior to November 2014 was performed using the Cochrane Library (Issue 9, 2014), PubMed (1997), EMBASE (1947) and Web of Science (1956). The Cochrane tool for risk of bias evaluation was used to assess the bias risk in the chosen data. A meta-analysis was conducted using RevMan 5.3. Of the 186 results, 14 RCTs, with a total of 659 participants from 11 countries, were included. Except for three studies assessed as having a ‘moderate risk of bias’, the RCTs were rated as having a ‘high risk of bias’. The methodological weaknesses were mainly due to ‘blinding’ and ‘allocation concealment’. The meta-analysis showed that diode LLLT significantly reduced orthodontic pain by 39 % in comparison with placebo groups (*P* = 0.02). Diode LLLT was shown to significantly reduce the maximum pain intensity among parallel-design studies (*P* = 0.003 versus placebo groups; *P* = 0.000 versus control groups). However, no significant effects were shown for split-mouth-design studies (*P* = 0.38 versus placebo groups). It was concluded that the use of diode LLLT for orthodontic pain appears promising. However, due to methodological weaknesses, there was insufficient evidence to support or refute LLLT’s effectiveness. RCTs with better designs and appropriate sample power are required to provide stronger evidence for diode LLLT’s clinical applications.

## Introduction

Pain and discomfort have long been among the most significant side effects of orthodontic treatment. An extensive prevalence of pain, ranging from 70 % in Caucasian populations to 95 % in Asian populations, has been reported in a large variety of orthodontic treatment modalities, including fixed and removable appliance therapy, separator and band placement, orthopaedic force application and even bracket de-bonding [1]. It has been well documented that orthodontic pain has a negative effect on patients’ quality of life. About half of patients have reported difficulties in physiological abilities such as chewing and biting following orthodontic treatment [2]. A longitudinal prospective study conducted by Zhang et al. showed that the oral health-related quality of life (OHQoL) of adolescents significantly deteriorated during fixed appliance treatment, with major manifestations in physical symptoms and functional limitations [3]. Liu et al. reported a similar finding among adult orthodontic patients [4]. Furthermore, surveys have shown that pain experience is a key barrier to the completion of treatment processes by orthodontic patients [5].

Despite the frequency of pain experience, insufficient evidence regarding the exact underlying mechanism has been obtained. Existing evidence shows that the application of orthodontic forces creates compression and tension zones in the periodontal ligament followed by a cascade of reactions: changes in blood flow, the release of inflammatory cytokines (prostaglandins, substance P, histamine, encephalin, leukotrienes, etc.), the stimulation of afferent A-delta and C nerve fibres, the release of neuropeptides and hyperalgaesia [6, 7].

Pain symptoms can be influenced by various factors, such as age, gender, psychological state, pain experience and cultural background, yet they progress in a similar pattern after the placement of orthodontic appliances [1]. Symptoms normally appear several hours after the force application, peak after 18–36 h and gradually decline to the baseline level within 7 days [8, 9].

Several treatment strategies have been suggested for the management of orthodontic pain, among which analgesics remain the major option. Non-steroidal anti-inflammatory drugs (NSAIDs) have been proven to be effective in pain control by inhibiting the cyclooxygenase enzyme system, leading to decreased synthesis of prostaglandins [10, 11]. However, the hindering of subsequent osteoclastic activity, causing reduced tooth movement rate, is a major concern for NSAIDs [12]. Moreover, common adverse effects, such as allergies, gastric ulcers and bleeding disorders, prevent the wide use of NSAIDs in clinical practice [10, 11]. Apart from medication, other methods, such as vibratory stimulation, chewing gum or a plastic wafer and transcutaneous electrical nerve stimulation, have been recommended for pain management [8, 13, 14]. However, the clinical application of such alternatives has been limited due to poor tolerance, unclear effects and scant evidence.

In recent years, low-level laser therapy (LLLT) has attracted increasing attention because of its unique advantages in analgesia, bio-stimulation and lack of adverse effects [15–18]. In contrast to high-powered surgical lasers, low-level lasers, also known as soft or low-intensity lasers, are classified as therapeutic lasers [17–19]. LLLT is defined as laser therapy with a low-energy output to keep the temperature of the treated tissue below 36.5 °C or normal body temperature [19]. Thus, compared to the utilisation of high-intensity lasers in cutting, ablation and thermal coagulation of tissue, low-level lasers have been demonstrated to have a non-thermal and biomodulative effect on the respiratory chain system within the membranes of mitochondria, triggering increased production of ATP, the ‘energy currency’ for cells [20]. This explains why LLLT have been shown to benefit wound healing and accelerate orthodontic tooth movement [15, 21]. Another important application of LLLT is for pain relief [16, 17]. However, the underlying mechanism remains unclear. LLLT has been reported to modify nerve conduction by affecting the synthesis, release and metabolism of various neurochemicals, including endorphins and encephalin [18]. It has also been postulated that the effects of LLLT on pain relief can be attributed to four aspects: inhibitory effects on nerve de-polarisation (especially C fibres), the reactivation of enzymes targeted at pain-inductive factors, the production of energy molecules (ATP) and the reduction of prostaglandin levels [22, 23].

Several types of low-level lasers have been found to have analgesic effects on pain caused by orthodontic mechanical stimuli, including the helium-neon laser, the carbon dioxide laser and the diode laser [24–26]. Introduced in 1980s, the relatively compact and low-cost diode laser, also known as a semi-conductive laser, has become the most widely used laser in dentistry. Based on its wavelength in the red and near-infrared region (600–1,000 nm), diode lasers can penetrate into deep tissues, promising desired effects on orthodontic pain control [18]. Moreover, diode laser devices offer greater optical efficiencies compared to its gas laser counterparts [17]. Two major types of low-level diode lasers, the GaAlAs laser (wavelength 780–890 nm) and the InGaAlP laser (wavelength 630–700 nm), have been used for orthodontic pain management [18]. In spite of the implicit merits of low-level diode lasers observed in a large number of clinical cases and trials, there is still no consensus on its exact analgesic effects because of inconsistent laser parameters, complex placebo effects and large inter-subject variations contributing to conflicting outcomes [17–19].

Although a few efforts have been made to assess the effect of LLLT on orthodontic pain management [27, 28], little attention has been paid to the specific effects of the most popular diode laser. Thus, a systematic review is essential for evidence-based clinical research and practice. This systematic review evaluated the effectiveness of diode LLLT on the management of pain induced by mechanical stimuli for orthodontic tooth movement based on outcomes from randomised controlled trials (RCTs).

## Materials and methods

This systematic review was performed with reference to the Cochrane Handbook for the Systematic Review of Interventions and the Preferred Reporting Items for Systematic Reviews and Meta-Analysis (PRISMA) [29, 30].

### Search strategy

An extensive literature research was conducted with the Cochrane Library (Issue 9, 2014), PubMed (1997), EMBASE (1947) and Web of Science (1956) for RCTs investigating the effect of diode LLLT on orthodontic pain without language limitations prior to November 2014. The reference lists of the retrieved articles were also reviewed. No additional hand searching of journals was performed. The search terms for orthodontic treatments consisted of ‘orthodontic’, ‘tooth movement’, ‘separator placement’, ‘archwire placement’, ‘canine retraction’ and ‘fixed appliance’; the search terms for the symptoms under investigation consisted of ‘pain’, ‘discomfort’ and ‘analgesia’, and these terms were combined with synonyms for LLLT, including ‘laser’, ‘laser therapy’, ‘laser irradiation’, ‘phototherapy’, ‘low-level laser’, ‘low-intensity laser’, ‘low-output laser’, ‘soft laser’, ‘semiconductor laser’, ‘diode laser’, ‘GaAlAs laser’ and ‘InGaAlP laser’.

### Eligible Criteria

Inclusion criteria are as follows:

1. The studies were RCTs examining the efficacy of diode LLLT on orthodontic pain control.
2. The participants received orthodontic treatment with mechanical forces directly exerted on the periodontal ligaments of the teeth (e.g. fixed appliance therapy, separator placement, etc.) There were no limitations on the age, gender, ethnicity and socio-economic status of the participants.
3. The participants were allocated to an experimental group or placebo/control group. The experimental group was treated with a low-level diode laser. The placebo group received a pseudo-laser application in identical settings without laser activation. No laser treatment was conducted on the control group.
4. The outcome variables included the prevalence, time course and intensity of pain assessed by means of a visual analogue scale (VAS) and/or questionnaires.

Exclusion criteria are as follows:

1. The literature was characterised as review articles, case reports, descriptive studies, opinion articles, abstracts, animal experiments or in vitro studies.
2. The participants had any systematic or dental diseases or were under medication that may have affected orthodontic tooth movement or pain perception.

Two reviewers screened the titles and abstracts of the studies independently. Subsequently, full-text reports were retrieved for all of the articles judged as potentially eligible or unclear due to insufficient information for a detailed evaluation. Cohen’s kappa test was used to assess the inter-reviewer reliability of the study selection, assuming 0.6 as an acceptable threshold value. Disagreements on the eligibility of studies were resolved by discussion between the two reviewers.

### Assessment of risk of bias

The assessment of the bias risk was conducted in accordance with the Cochrane Tool for risk of bias assessment [29]. The methodological quality of each included study was judged with respect to the risk status (‘low’, ‘unclear’ and ‘high’) in seven domains, covering bias in selection, performance, detection, attrition, reporting and other aspects. The comprehensive methodological quality of a study was classified as low risk of bias (six domains assessed as ‘low risk’), moderate risk of bias (one or more domains assessed as ‘unclear risk’) and high risk of bias (one or more domains assessed as ‘high risk’).

### Extraction of data

The following information was extracted from the included studies: the randomisation method, allocation concealment, blinding, study design, demographic features, sample size, lost to follow-up, orthodontic treatment approach, laser parameters and regimen, outcome measurements, adverse effects, assessment interval and follow-up duration.

### Statistical analysis

The meta-analysis was conducted using RevMan 5.3. The mean difference (MD) with a 95 % confidence interval (CI) was adopted for continuous data, such as the VAS score and time course of pain. To assess the intervention effect on the maximum and mean pain intensity, the generic inverse variance method was applied to the combined data from studies with parallel designs and split-mouth designs [31]. In cases for which the standard error (SE) of the effect estimate was not available or not calculable from the raw data, the method of variance imputation was used to estimate the variance values [29]. Because one study only presented the MD and SE of a paired comparison, the generic inverse variance method was also applied to estimate the effect on the termination of pain [32]. The intervention effect based on a dichotomous outcome (prevalence of pain) was measured by the relative risk (RR) with a 95 % CI. The heterogeneity of the data was assessed by *I* ^2^ statistics at *α* = 0.10. A random-effects model was applied if substantial heterogeneity was detected (*I* ^2^ > 50 %), otherwise a fixed-effects model was used. The statistically significant level for the hypothesis test was set at *α* = 0.05 for two-tailed *z* tests. A subgroup analysis was conducted with respect to different study designs (split-mouth or parallel design), if possible.

## Results

### Search results

**Fig. 1:**
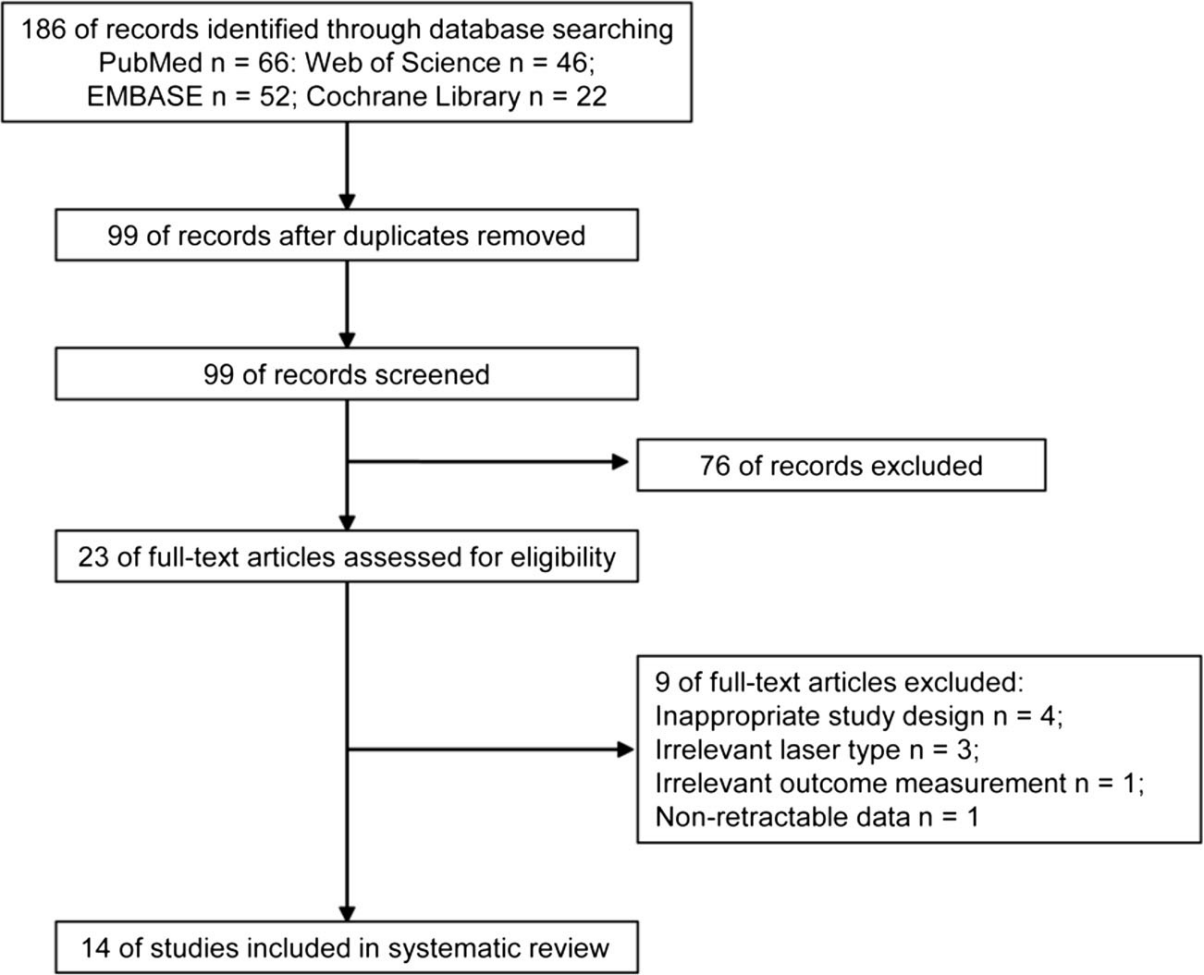
PRISMA flow diagram of the study inclusion process

Initially, 186 studies were identified through the electronic search, of which 99 studies remained after removing duplicates. During the first stage, 76 studies were excluded based on the evaluation of the titles and abstracts (inter-reviewer agreement, kappa = 0.91). In the second stage, after screening the full-text articles of the remaining 23 studies, a total of 14 eligible studies were included for the systematic review (inter-reviewer agreement, kappa = 0.94) [36–49]. The whole selection process is shown in Fig. 1.

### Characteristics of included studies

The included studies were conducted in 11 countries with sample sizes varying from 12 to 120 and participants’ ages ranging from 11 to 33 years. Among the 14 studies, 9 used a split-mouth design, whereas the rest used a parallel design. The most commonly used model to trigger orthodontic pain appeared to be separator placement, followed by canine retraction and archwire placement (Table 1). The majority of studies used a GaAlAs diode laser, with a wavelength between 800 and 830 nm. However, the output power and energy varied greatly among studies (0.18–9 J per treatment point). The application methods of the diode laser were also diversified among the studies. Most studies irradiated several points along or surrounding the root with direct contact between the laser tips and the alveolar mucosa. A single-application method was observed in about half of studies, whereas for multiple-application approach, additional irradiations were typically applied within 1 week after the orthodontic treatment (Table 2). With regard to the evaluation method, almost all of the studies used a VAS for measuring pain intensity. Several studies also used self-designed questionnaires to investigate the time course of pain. The most frequently applied follow-up period was 7 days after the force application, which coincided with the commonly reported progress pattern of pain (Table 1).

**Table 1:** Characteristics of included studies

Study ID	No. (M/F)	Country	Age in mean ± SD (range)	Study design	Grouping method	Orthodontic treatment	Evaluation method	Evaluation interval
Eslamian et al. [33]	37 (12/25)	Iran	24.97 (11–32)	Split-mouth	I: *N* = 37 P: *N* = 37	Separator placement	VAS	Pre-LLLT, 6, 24, and 30 h, day 3, 4, 5, 6 and 7 post-LLLT
Heravi et al. [34]	20 (3/17)	Iran	22.1 ± 5.3 (15–31)	Split-mouth	I: *N* = 20 P: *N* = 20	Canine retraction	VAS	Day 0, 4, 7, 11, 15, 28, 32, 35, 39, 43 and 56 (pre-LLLT)
Abtahi et al. [35]	29 (24/5)	Iran	15.03 (12–22)	Split-mouth	I: *N* = 29 P: *N* = 29	Separator placement	VAS	Pre- and post-LLLT for 5 days
Artés-Ribas et al. [36]	20 (6/14)	Spain	26.4 (19–33.8)	Split-mouth	I: *N* = 20 P: *N* = 20	Separator placement	VAS	Pre-LLLT, 5 min, 6 h, 24 h, 48 h and 72 h post-LLLT
Domínguez and Velásquez [37]	59 (19/40)	Colombia	24.3 ± 3	Split-mouth	Self-ligation group I: *N* = 29, P: *N* = 29 Straight-wire group I: *N* = 30, P: *N* = 30	Archwire placement (0.019 × 0.025 in. stainless steel)	VAS	2 h, 6 h, 24 h, day 2, 3 and 7 post-LLLT
Bicakci et al. [38]	19 (8/11)	Turkey	13.9 (13.5–14.5)	Split-mouth	I: *N* = 19 P: *N* = 19	Molar band placement	VAS	5 min, 1 h and 24 h after placement
Doshi-Mehta and Bhad-Patil [39]	20 (8/12)	India	12–23	Split-mouth	I: *N* = 30 P: *N* = 30	Canine retraction	VAS	Day 1 after placement, Day 3 and 30
Angelieri et al. [40]	12	Brazil	12.66	Split-mouth	I: *N* = 12 P: *N* = 12	Canine retraction	VAS	12, 24, 48 and 72 h post-LLLT and repeat in the 2nd month
Lim et al. [41]	39	Singapore	21-24	Split-mouth	15 s group: *N* = 39 30 s group: *N* = 39 60 s group: *N* = 39 P: *N* = 39	Separator placement	VAS	Day 0 (pre-separation, pre-LLLT and post-LLLT); day 2, 3, 4 and 5 (pre-LLLT and post-LLLT)
Kim et al. [42]	88 (23/65)	Korea	22.7	Parallel	I: *N* = 28 P: *N* = 30 B: *N* = 30	Separator placement	VAS	5 min, 1, 6, 12 h and day 1, 2, 3, 4, 5, 6 and 7 after placement
Marini et al. [32]	120 (64/56)	Italy	23.01 ± 1.39	Parallel	I: *N* = 40 P: *N* = 40 B: *N* = 40	Separator placement	VAS A modified version of Harazaki’s questionnaire	Immediately and 12, 24, 36, 48, 72 and 96 h after placement
Nobrega et al. [43]	60 (22/38)	Brazil	12–26	Parallel	I: *N* = 30 P: *N* = 30	Separator placement	VAS	2, 6 and 24 h, day 3 and 5 after placement
Tortamano et al. [44]	60 (18/42)	Japan	15.9 (12–18)	Parallel	I: *N* = 20 P: *N* = 20 B: *N* = 20	Archwire placement (0.014 in. stainless steel)	VAS Harazaki’s questionnaire	Over the next 7 days
Turhani et al. [45]	76 (30/46)	Austria	23.1	Parallel	I: *N* = 38 P: *N* = 38	Archwire placement (0.016 in. stainless steel)	Self-designed questionnaire	6, 30 and 54 h after treatment

*No.* number of participants, *M* male, *F* female, *I* intervention group, *P* placebo group, *B* blank control group, *VAS* visual analogue scale

**Table 2:** The parameters and regimen of diode laser applied in included studies

Study ID	Type of laser	Wavelength	Output/energy (density)	Total dose per point (tooth)	Time of exposure	Method of application	Frequency of laser treatment
Eslamian et al. [33]	GaAlAs laser, continuous mode	810 nm	100 mW, 2 J/cm^2^	2 J/point, 20 J/tooth	200 s/tooth	Perpendicular to the long axis of the teeth on 5 points of the buccal and lingual side (10 points/tooth)	Immediately after separator placement and 24 h later
Heravi et al. [34]	GaAlAs laser, continuous mode	810 nm	200 mW, 21.4 J/cm^2^	6 J/point, 60 J/tooth	300 s/tooth	Perpendicular in contact with the mucosa on 5 points of the buccal and lingual side (10 points/tooth)	Day 0, 4, 7, 11, 15, 28, 32, 35, 39, 43 and 56
Abtahi et al. [35]	GaAs laser, high pulse mode	904 nm	200 mW	1.5 J/point, 6 J/tooth	30 s/tooth	Perpendicular in contact with the gum on 2 points of the vestibular and lingual side (4 points/tooth)	Immediately after separation and once daily for the following 4 days
Artés-Ribas et al. [36]	GaAlAs laser, continuous mode	830 nm	100 mW, 5 J/cm^2^	2 J/point, 12 J/tooth	120 s/tooth	In contact with the mucosa on 3 points of the buccal and palatal side (6 points/tooth)	Single application (immediately after separator placement)
Domínguez and Velásquez [37]	GaAlAs laser, continuous mode	830 nm	100 mW, 80 J/cm^2^	2.2 J/area, 4.4 J/ tooth	44 s/tooth	Scanned 1 mm from the mucosa along the vestibular and palatal surface of the root (2 areas/tooth)	Single application
Bicakci et al. [38]	GaAlAs laser, continuous mode	820 nm	50 mW, 7.96 J/cm^2^	0.25 J/point, 1 J/ tooth	20 s/tooth	In direct contact on 4 points around the tooth (4 points/tooth)	Immediately after band placement and 24 h later
Doshi-Mehta and Bhad-Patil [39]	GaAlAs laser, continuous mode	800 nm	100 mW	0.8 J/point, 8 J/tooth	80 s/tooth	In direct contact on 5 points of the buccal and lingual side (10 points/tooth)	Day 0, 3, 7 and 14 in the 1st month, every 15th day until complete canine retraction on the laser side
Angelieri et al. [40]	ArGaAl laser	780 nm	20 mW, 5 J/cm^2^	0.2 J/point, 2 J/tooth	100 s /tooth	Perpendicular in contact with the mucosa on 5 points of the buccal and lingual side (10 points/tooth)	Immediately after spring activation, day 3 and 7
Lim et al. [41]	GaAsA1 laser, continuous mode	830 nm	30 mW	0.45, 0.9, 1.8 J/tooth	15, 30 and 60 s/tooth	Applied onto the buccal mucosa overlying the middle third of the root (1 point/tooth)	Immediately after separator placement and the following 4 days
Kim et al. [42]	AlGaInP laser	635 nm	6 mW, 10 mJ	0.18 J/point, 0.72 J/tooth	120 s/tooth	In direct contact with the mucosa on 2 areas of the buccal and lingual side (4 points/tooth)	Immediately after separator placement and every 12 h for 1 week
Marini et al. [32]	GaAs laser, superpulse mode	910 nm	160 mW	9 J/point, 18 J/tooth	113 s/tooth	Applied on the cervical third of buccal and lingual gingiva (2 points/tooth)	Single application (immediately after separator placement)
Nobrega et al. [43]	GaAsA1 laser	830 nm	40.6 mW, 1 or 2 J/cm^2^	5 J/tooth	125 s/tooth	Applied on root apex and along the root axis on the buccal side (4 points/tooth)	Single application (immediately after separator placement)
Tortamano et al. [44]	GaAlAs laser, continuous mode	830 nm	30 mW, 0.5 J/cm^2^	0.48 J/point, 4.8 J/tooth	160 s/ tooth	Applied on 5 areas of the buccal and lingual mucosa overlying the dental root (10 points/tooth)	Single application (immediately after archwire placement)
Turhani et al. [45]	Diode laser continuous mode	670 nm	75 mW, 4.2 J/cm^2^	2.25 J/tooth	30 s/tooth	At a distance of 5 to 8 mm with a right angle to the mucosa at the level of the biomechanical centre of resistance (1 point/tooth)	Single application (immediately after archwire placement)

### Assessment of methodology quality

**Fig. 2:**
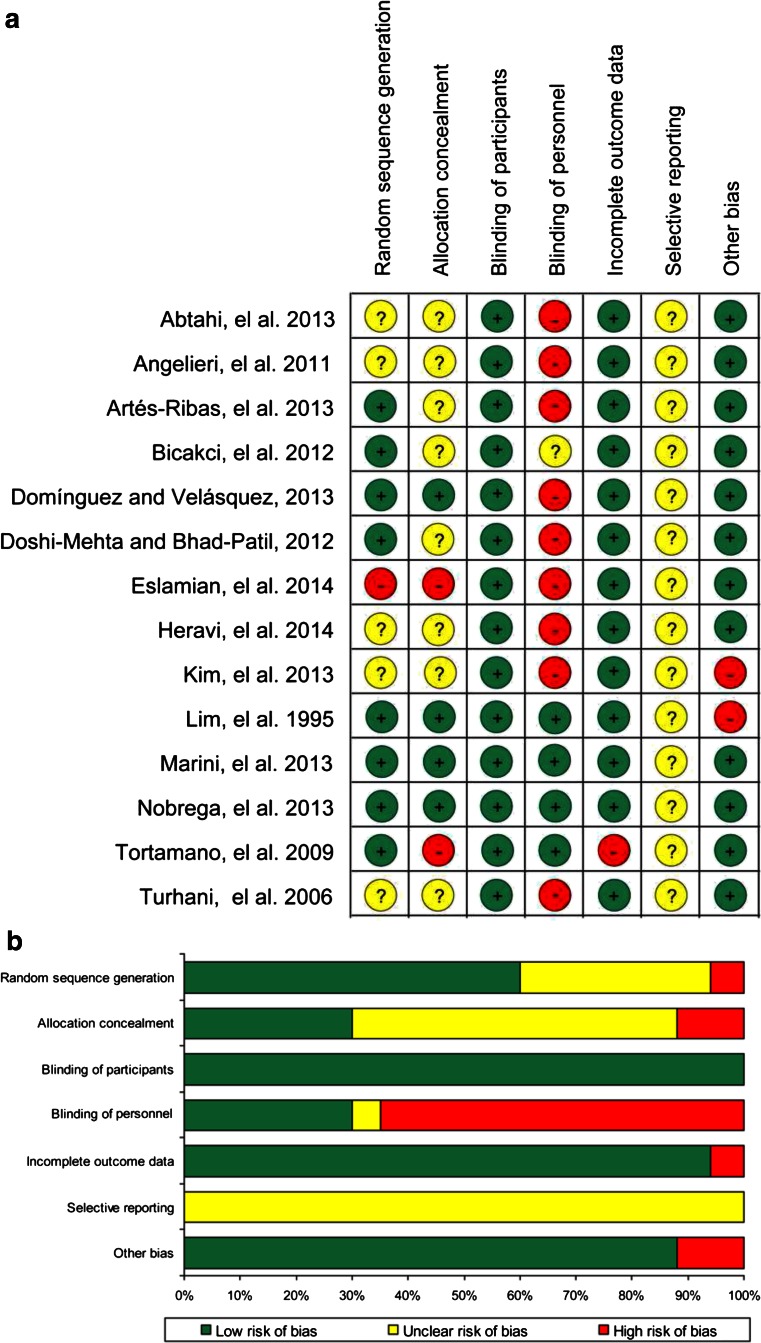
**a** Risk of bias summary: review authors’ judgments about each risk of bias item for each included study. **b** Risk of bias graph: review authors’ judgments about each risk of bias item presented as percentages across all included studies

The results of the methodological quality assessment were shown in Figs. 2a, b. Of the 14 included studies, only 3 were assessed as having a moderate risk of bias, whereas the rest all implied a high risk of methodological drawbacks [32, 38, 43]. Among all seven domains, ‘blinding of key personnel’ accounted for the principal risk factor affecting methodology quality. Only four studies reported that a double-blind method was used to prevent participants and key personnel from perceiving the assignment to diode LLLT or placebo (control) [32, 41, 43, 44]. One study failed to explicitly mention the blinding measure adopted in the experiment and assessment process [38]. However, the majority of studies applied a single-blind method, in which the participant was blinded and the operator who performed the intervention was aware of the grouping information. Although all of the studies were presented as randomised, one study used an inadequate sequence generation method [33]. The most commonly used randomisation methods were based on computer programs [37, 38, 43] and random number tables [36, 44]. Three trials used block randomisation to ensure a balance in the assignments to the experimental or placebo (control) groups [32, 39, 43]. One study used the Latin Square method for randomisation [41]. Another key risk factor was that most studies failed to state which method they used to conceal the allocation sequence, except four studies [32, 37, 41, 43]. Moreover, one study presented incomplete outcome data without adequately addressing the missing information [44]. Apart from these clearly defined categories of bias risk, one trial recruited participants among dental students, limiting the generalisation of the conclusion to the entire population [41]. The laser was applied by the participants instead of by a well-trained clinician, suggesting a risk of bias induced by a potential inconsistency in intervention [42]. None of these included studies provided sufficient information for the judgement of ‘selective outcome reporting’.

### Effect of diode LLLT on orthodontic pain control

#### Prevalence of pain

**Fig. 3:**
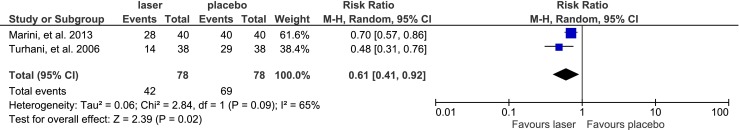
Comparison: laser versus placebo, outcome: prevalence of pain (studies with parallel design)

Two studies reported the detailed number of participants experiencing pain after orthodontic treatment, enabling a synthesising of the data by meta-analysis. The effect of the intervention was presented with a forest plot (Fig. 3). It was shown that diode LLLT reduced the prevalence of orthodontic pain by 39 % at a significant level compared with the placebo group (RR = 0.61, 95 % CI range: 0.41 to 0.92, *P* = 0.02; *χ* ^2^ = 2.84, *P* = 0.09, *I* ^2^ = 65 %).

#### End of pain

**Fig. 4:**
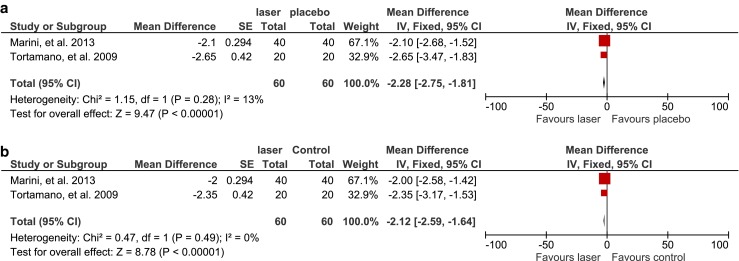
**a** Comparison: laser versus placebo, outcome: end of pain (studies with parallel design). **b** Comparison: laser versus control, outcome: end of pain (studies with parallel design)

The time course of pain was investigated in two studies via questionnaires modified from that used by Harazaki, providing continuous data for the meta-analysis of the endpoint of pain (Fig. 4a, b). A forest plot revealed that pain subsided significantly earlier in the laser-irradiated group compared with the placebo group (MD = −2.28, 95 % CI range −2.75 to −1.81, *P* < 0.00001), with insignificant heterogeneity in the data (*χ* ^2^ = 1.15, *P* = 0.28, *I* ^2^ = 13 %). The comparison of the laser-treated versus control groups showed a similar pattern (MD = −2.12, 95 % CI range −2.59 to −1.64, *P* < 0.00001; *χ* ^2^ = 0.47, *P* = 0.49, *I* ^2^ = 0).

#### Pain intensity

**Fig. 5:**
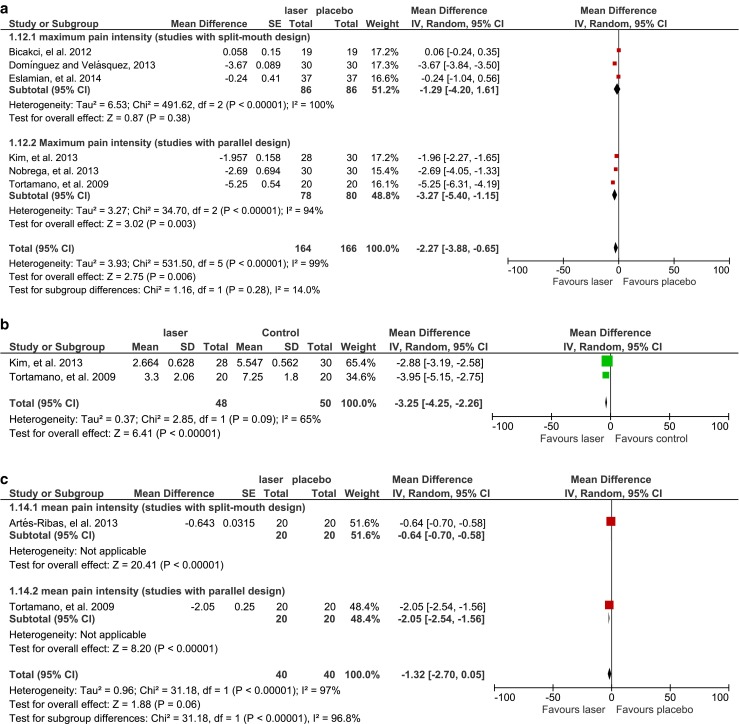
**a** Comparison: laser versus placebo, outcome: maximum pain intensity, subgroup analysis: split-mouth versus parallel design. **b** Comparison: laser versus control, outcome: maximum pain intensity (studies with parallel design). **c** Comparison: laser versus placebo, outcome: mean pain intensity, subgroup analysis: split-mouth versus parallel design

Adequate continuous data concerning the most severe pain level measured with a VAS score was available in six studies, which were further divided into two subgroups according to different study designs (split-mouth and parallel designs) for meta-analysis (Fig. 5a). The assessment of split-mouth design studies showed that compared to placebo groups, the maximum pain intensity slightly decreased as a result of diode LLLT, but the result was not statistically significant (MD = −1.29, 95 % CI range −4.20 to 1.61, *P* = 0.38; *χ* ^2^ = 491.62, *P* < 0.00001, *I* ^2^ = 100 %). In contrast, diode LLLT was shown to significantly reduce the peak pain level by 3.27 compared with placebo groups in parallel-design studies (MD = −3.27, 95 % CI range −5.40 to −1.15, *P* = 0.003; *χ* ^2^ = 34.70, *P* < 0.00001, *I* ^2^ = 94 %). However, no significant difference was detected among subgroups (*χ* ^2^ = 1.16, *P* = 0.28, *I* ^2^ = 14 %). Only the parallel-design studies provided adequate data for comparisons with control groups (Fig. 5b). Diode LLLT demonstrated a statistically significant advantage in reducing the maximum pain intensity (MD = −3.25, 95 % CI range −4.25 to −2.26, *P* < 0.00001; *χ* ^2^ = 2.85, *P* = 0.09, *I* ^2^ = 65 %).

Only two studies calculated the mean pain intensity experienced by participants during follow-ups (Fig. 5c). One study used a split-mouth design, whereas the other applied a parallel design. Both studies showed a significant reduction of the mean pain intensity in the groups treated with diode LLLT compared with the placebo groups (MD = −0.64, 95 % CI range −0.70 to −0.58, *P* < 0.00001, for the split-mouth design study; MD = −2.05, 95 % CI range −2.54 to −1.56, *P* < 0.00001, for the parallel-design study). However, only a marginal difference was detected in the overall assessment, slightly favouring the diode LLLT group (MD = −1.32, 95 % CI range −2.70 to 0.05, *P* = 0.06; *χ* ^2^ = 31.18, *P* < 0.00001, *I* ^2^ = 97 %).

#### Adverse events

All of the included studies described that both the patients and therapists wore specially designed protective goggles to avoid potential harm of irradiation to their eyes. No adverse events were reported.

## Discussion

After an extensive search and careful selection, a total of 14 RCTs with divergent study methodologies and laser dosimetry were included in a qualitative review. The assessment of methodology quality showed a high risk of bias in 11 RCTs, indicating a notable under-grading of the quality of the existing evidence. A quantitative analysis was conducted to evaluate the effects of diode LLLTs on the prevalence, time course and intensity of orthodontic pain. Diode LLLT was shown to be beneficial to the reduction of pain prevalence and to the termination of pain, which agreed with the conclusions of previous systematic reviews on the analgesic effects of LLLT [27, 28]. Nevertheless, LLLT’s effectiveness in decreasing pain intensity was clouded by the differences in the study designs. Notably, there was extensive methodological weakness and substantial heterogeneity across almost all domains of meta-analysis. Thus, there was insufficient evidence to draw a conclusion on whether diode LLLT was an effective treatment strategy for orthodontic pain control. In general, there were three major factors contributing to the weakness of the existing evidence: study methodology, individual variation and laser dosimetry.

### Effects of study methodology on outcome

The presence of paired or multiple organs (arches, quadrants, teeth) in oral cavities suggests a split-mouth design, in which alternative treatments (no less than two interventions) are applied to different sections (teeth, tooth surfaces) of the same patient’s mouth [46]. Compared with parallel designs, in which each individual only receives one intervention, split-mouth designs can achieve meaningful results with a relatively smaller sample size. In addition, the effects of inter-subject variation can be minimised when the individual is self-matched or self-controlled [31, 46]. This characteristic makes split-mouth designs particularly appropriate for studies assessing highly subjective outcomes, such as pain perception. The decision on whether to choose a split-mouth design depends on the nature of the disease and treatment effect [46]. Low-level laser therapy appears to have a localised effect on orthodontic pain, which is a relatively stable and uniformly distributed symptom [18]. Only a few studies have reported systematic effects of LLLT on wound healing; however, the evidence was limited by unclarified mechanisms [47]. Thus, we consider that the application of split-mouth designs to studies investigating orthodontic pain is justified and advantageous compared to parallel-design studies. The high heterogeneity among the included studies was due to different study designs, to a great extent. According to the recommendations by Lesaffre et al. and the Cochrane Oral Health group, split-mouth and parallel-arm studies should be assessed and interpreted separately [29, 31]. However, limited evidence was found in previous reviews addressing the association between study designs and effect estimates. Therefore, we assessed the effects of diode LLLT by analysing these two types of designs independently. The subgroup analysis revealed differences in diode LLLT’s effects on pain intensity, with studies of split-mouth design showing less statistically significant effects. However, the difference failed to reach a significant level, in accordance with the conclusion of Smaïl-Faugeron et al. [48].

It is noteworthy that the quality of the evidence was greatly affected by defections in methodology and inconsistencies in laser dosimetry among the limited number of studies. Most studies were implemented without effective blinding of the intervention operators and outcome assessors. Moreover, appropriate measures to avoid foreseeing the intervention method were neglected in the majority of the studies. Besides, one study with a split-mouth design adopted an inadequate method of randomisation [33], whereas another five studies did not describe the method explicitly [34, 35, 40, 42, 45]. Methodological drawbacks existed extensively in both study designs, affecting the reliability of the conclusions.

In addition, the orthodontic mechanical stimuli used to trigger pain varied among the included studies. The placement of a separator was applied most frequently as a model to stimulate orthodontic pain. However, there can be differences in pain response and intensity between that induced by a separator (single tooth) and by an archwire (entire arch). Moreover, the laser dosimetry and application method also differed according to various experimental models, affecting the comparability among studies. Thus, future research is advisable to adopt a common model for assessing diode LLLT’s effects on orthodontic pain that is closer to the real circumstances during orthodontic tooth movement.

Consisting of a marked horizontal line from 0 cm (no pain) to 10 cm (worst pain possible), the VAS is recognised as a sensitive and reliable instrument for evaluating an individual’s subjective feeling of pain level quantitatively, superior to the verbal categorical rating scale (VRS) [49]. Almost all of the included studies applied the VAS to assess orthodontic pain, ensuring the reliability and comparability of outcomes. Several studies with parallel designs also incorporated questionnaires, which helps in understanding the effect of diode LLLT on the progression pattern of pain. However, there were no acknowledged guidelines on the questionnaire design and limited data could be extracted from studies with a split-mouth design, disqualifying the synthesis of the outcomes.

### Effects of laser dosimetry on outcome

Another important issue in this field is that there is no current consensus on the optimal parameters of diode low-level lasers. The efficacy of diode LLLT can be determined by a combination of multiple factors, including the light source, wavelength, spot-size, mean output measured in watts, energy measured in Joules, mode of operation (continuous wave or pulsed), application interval and frequency [19]. It is recognised that a therapeutic window for diode LLLT exists. Irradiation energy exceeding this range will cause photobioinhibitory effects, whereas an extremely low dosage is not sufficient to trigger the desired biological effects. However, the exact dose range remains controversial, since there is a great variation in study designs and laser parameters among previous research [32–45]. Kert and Rose recommended a treatment strategy of applying a diode low-level laser in a continuous mode, with energy between 0.5 and 10 J per treatment point and in contact with the tissue surface for deeper effects [19]. Some researchers have also suggested using 2–4 J per treatment point with multiple applications at the beginning of the treatment [18]. Among the included studies, the parameters of the diode laser varied greatly with respect to the wavelength (635–910 nm), output power (6–200 mW), energy (0.18–9 J per treatment point), application method (treatment points and contact mode) and treatment interval. This can partly explain the significant heterogeneity among studies during the assessment of the intervention effects. However, subgroup analysis and meta-regression to compare the effects of diode LLLT with different parameters was disqualified due to the confounding heterogeneity in dosimetry and insufficient numbers of studies. It should be noted that there was no standard in the reporting of laser parameters among the studies. Important information such as beam size and energy density was missing in several studies, making comparisons and generalisations difficult.

### Effects of individual variation on outcome

Furthermore, considerable inter-subject variation may have contributed to the conflicting results. It has been reported that the perception of orthodontic pain can be affected by various factors such as age, gender, emotional status, past pain experience and so on [1]. Turhani et al. reported a smaller difference in pain intensity between laser and placebo groups among patients over 18 years old compared with those under 19. They also found that women appeared to recover more quickly than men under laser therapy, suggesting variations in the effects of diode LLLT among different populations [57]. Considering the wide age range (11–33 years old) and gender distribution among the included studies, there were substantial differences in the selection of the study sample. However, instead of assessing the analgesic effect of diode LLLT separately based on group characteristics, most studies pooled all data and analysed the overall effect. In addition, it is necessary to conduct sample size calculations based on data provided by the pilot study or previous literature to ensure sufficient test power.

### Suggestions to future research

In view of the weakness of the current evidence, the following strategies are suggested to improve the overall quality of related clinical trials. First, well-designed RCTs should be conducted with reference to Cochrane’s risk of bias assessment criteria. Adequate randomisation methods, effective allocation concealment and blinding measures should be adopted in the design of a RCT to ensure outcome reliability and minimise placebo effects. Moreover, an appropriate method of addressing missing data should be explicitly described. Second, split-mouth designs should be recommended on the premise that no carry-over effects of diode LLLTs in orthodontic pain relief are verified. However, stricter requirements on study and statistical methodology are expected in RCTs of this design. Apart from the examination of pain intensity, more attention should be paid to the effects of diode LLLT on the progression pattern of pain, based on questionnaires designed according to pre-specified standards. Third, a consensus should be made on the range of potentially effective dosimetry of diode LLLTs, followed by a test of its effectiveness in vitro and subsequently in vivo. It is essential to report the laser parameters in adherence to recognised criteria, as suggested by some researchers and organisations [50]. Additionally, appropriate sample selections and assessment methods should be taken into account when investigating diode LLLT’s analgesic effects on a specific target population.

## Conclusion

There is insufficient evidence to support or refute the effectiveness of diode LLLT for orthodontic pain management. Despite the extensive methodological weakness and significant heterogeneity of existing evidence, diode LLLT has demonstrated benefits in reducing the prevalence of and inducing the earlier termination of orthodontic pain; diode LLLTs also exhibit some effects on decreasing pain intensity. Further research with a better study design, appropriate sample power and controlled laser dosimetry is required to provide more reliable evidence for the clinical application of diode LLLT.
